# Informal caregiver burden in middle-income countries: Results from Memory Centers in Lima – Peru

**DOI:** 10.1590/S1980-57642014DN84000012

**Published:** 2014

**Authors:** Nilton Custodio, David Lira, Eder Herrera-Perez, Liza Nuñez del Prado, José Parodi, Erik Guevara-Silva, Sheila Castro-Suarez, Marcela Mar, Rosa Montesinos, Patricia Cortijo

**Affiliations:** 1 Servicio de Neurología, Clínica Internacional, Lima, Peru; 2 Unidad de Diagnóstico de Deterioro Cognitivo y Prevención de Demencia, Clínica Internacional, Lima, Peru; 3 Unidad de Investigación, Instituto Peruano de Neurociencias, Lima, Peru; 4 Unidad de Diseño y Elaboración de Proyectos de Investigación, Lima, Peru; 5 Centro de Investigación para el Desarrollo Integral y Sostenible (CIDIS), Universidad Peruana Cayetano Heredia, Lima, Peru; 6 Servicio de Neurología. Clínica Maisson de Sante, Lima, Peru; 7 Centro de Investigación del Envejecimiento, Facultad de Medicina Humana, Universidad San Martín de Porres, Lima, Peru; 8 Departamento de Medicina, Hospital de Chancay, Lima, Peru; 9 Servicio de Neurología de la Conducta, Instituto Nacional de Ciencias Neurológicas, Lima, Peru; 10 Servicio de Medicina Física y Rehabilitación, Clínica Internacional, Lima, Peru

**Keywords:** dementia, caregiver, burden, Peru

## Abstract

**Objective:**

The aim of this study was to evaluate caregiver burden based on Zarit Burden Interview (ZBI) and depression in caregivers on the Beck Depression Inventory-II (BDI-II).

**Methods:**

Literate individuals, 18 years or older, who spoke Spanish as their native language were included. Demographic characteristics: Age, sex, education, relationship to person with dementia, length of time caregiving, other sources of help for caring, impact on the household economy, family support, and perception of impaired health; and Clinical data on care-recipients: type of dementia, time since diagnosis, treatment, and Global Deterioration Scale (GDS); the ZBI and BDI-II. Descriptive and analytical statistics were employed to assess caregiver burden and predictors of higher burden in caregivers.

**Results:**

A total of 92 informal caregivers were evaluated. Regarding care-recipients, 75% were 69 years old or over, 75% had at least one year since diagnosis, 73.9% had Alzheimer's disease, 84.8% received treatment, 75% scored 5 or over on the GDS. For caregivers, 75% were 55.5 years old or over, predominantly female (81.5%), married (83.7%), the spouse of care-recipients (60.87%), had at least 10 years of education (75.0%) and one year of caregiving (75%), reduced entertainment time (90.2%) and self-perception of impaired health (83.7%). Median score on the ZBI was 37.5 (minimum value = 3; and maximum value = 74). The coefficient of BDI was 1.38 (p-value <0.001).

**Conclusion:**

This sample of Peruvian informal caregivers showed elevated ZBI values. Self-perception of worsened health, repercussion on the family economy and time caregiving were the main determinants of ZBI, although only BDI was a consistent predictor of ZBI.

## Abstract (pt)

**Objetivo:**

Avaliar a sobrecarga do cuidador baseada na entrevista de Zarit (ZBI).

**Métodos:**

Nós incluímos cuidadores com 18 anos ou mais de idade, língua espanhola como nativa, alfabetizados. Foram avaliadas características demográficas: idade, gênero, educação, relação com o paciente com demência, extensão do cuidado, outras fontes de ajuda para os cuidados, impacto na economia doméstica, suporte familiar e percepção do comprometimento de saúde; e dados clínicos de saúde dos pacientes: tipo de demência, tempo desde o diagnóstico, tratamento e escala de deterioração global (GDS); ZBI e BDI-II.

**Resultados:**

Avaliamos 92 cuidadores informais. Os pacientes tinham 69 anos ou mais, 75% tinha pelo menos um ano do diagnóstico, 73,9% com doença de Alzheimer, 84,8% recebia algum tratamento, 75% tiveram GDS de 5 ou mais. Quanto aos cuidadores: 75% tinham 55 anos ou mais, mulheres (81,5%), casados (83,7%), cônjuge (60,9%), com pelo menos 10 anos de educação (75%) e um ano de cuidado (75%), tempo reduzido de entretenimento (90,2%) e autopercepção de comprometimento da saúde (83,7%). A mediana da ZBI foi 38 (mínimo: 3 e máximo: 74). O coeficiente de BDI foi 1,38 (p<0,001).

**Conclusão:**

Nossa amostra de cuidadores informais peruanos mostram valores consideráveis na ZBI. A autopercepção de piora de saúde, repercussão na economia familiar e tempo de cuidado foram os principais determinantes da ZBI, embora somente a BDI foi um preditor consistente de ZBI.

## INTRODUCTION

Providing care to a relative with functional or cognitive needs is associated with negative consequences for the caregiver, especially if the care-recipient has dementia.^1-4^ Furthermore, these negative consequences for caregivers indirectly impact the health of care-recipients. Thus, the caregiver's status should be measured in all intervention studies of dementia, including clinical drug trials.^5^

Several measures have been used for assessing the consequences of caregiving, such as socioeconomic impact, health status, quality of life, and, ultimately, burden.^6-10^ Burden has been conceptualized as the set of objective and subjective problems that may be experienced by a caregiver.^11^ The objective problems include activities (time and care tasks), facts (effects on physical and psychological health), and events (social, economic, and occupational impacts). The attitudes and emotional reactions of caregivers (guilt, stress and concerns) are the subjective problems associated to the experience of care.^12^ The majority of the tools for measuring caregiving focus only on the subjective dimension of burden.

Currently, there are several tools for assessing caregiver burden,^13-22^ but their utility is variable because their conceptualization is heterogeneous.^23^ Zarit developed a scale for assessing the subjective burden associated with functional and cognitive impairment of demented patients living in the community.^22^ The Zarit Burden Interview (ZBI), a brief and simple scale, is the most used tool for measuring the global status of informal caregivers, identifying critical areas, and evaluating the efficacy of applied interventions.^23^ This scale has been validated for various languages, including Spanish.^2,24-29^

The aim of the study was to assess the burden in Peruvian informal caregivers of patients with dementia.

## METHODS

**Study design.** This was a cross-sectional study in a sample of caregivers from private memory centers.

**Population and sample.** Participants were informal caregivers of patients with an established diagnosis of dementia. Caregivers 18 years or older, who spoke Spanish as their native language, were literate, consecutively enrolled during the period spanning from June to August 2014, were included. Three recruitment centers were involved:

[1] Instituto Peruano de Neurociencias;
[2] Clínica Internacional - Lima; and
3) Clínica Internacional - San Borja.

Based on previous studies, informal caregiver was defined as the person who identified themselves as the primary unpaid caregiver for the person with dementia. The person with dementia lived in the community, and caregivers reported that more than 1 hour of their day was devoted to caregiving duties, and that they had been providing care for more than three consecutive months.^13,14^

**Procedures.** The authors extended the invitations to participate. All the caregivers were evaluated using the same instruments (questionnaire and tests). The clinical data of patients were verified from medical records.

**Test of interest.** The ZBI is a screening test for assessing caregiver burden, which has been specially designed to reflect the burden experienced by caregivers of dementia patients. It can be completed by caregivers themselves or as part of an interview. Caregivers are asked to answer a series of 22 questions about the impact of the patient's disabilities on their life. For each item, caregivers have to indicate how often they felt this way (never, rarely, sometimes, quite frequently, or nearly always).^22,30^

The ZBI include three subtests:

[1] Consequences of care on caregiver;
[2] Beliefs and expectations about ability to care; and
[3] Relationship between caregiver and patient. This test is scored by adding the numbered responses of the individual items. For each option mentioned "(never, rarely, sometimes, quite frequently, nearly always)", a score is assigned in the 0-4 range and maximum score is 88.

Higher scores indicate greater caregiver burden. Additionally, estimates of the degree of burden can be made as following:

[1] Little or no burden if ZBI ≤ 20;
[2] Mild to moderate burden if ZBI > 20 and ZBI ≤ 40;
[3] Moderate to severe burden if ZBI > 40 and ZBI ≤ 60; and
[4] Severe burden if ZBI > 60.^22,30,31^

**Other measures and tests.** *Demographic characteristics*. The following characteristics were assessed: age (persons with dementia and caregivers), sex, education, relationship to person with dementia, length of time caregiving, and existence of other sources of help for caring (caregivers). Additionally, caregivers were asked to report working status, impact on the household economy, existence of family support, and perception of impaired health.

*Depression in caregivers*. Depressive symptomatology was measured by the Beck Depression Inventory-II (BDI-II) test, a widely used self-report measure for assessing this problem in community samples.^32^ The BDI-II has shown high performance in discriminating between depressed and non-depressed subjects among older adults.^33,34^ The instrument validity appears to be independent of language.^35^ The scale contains 20 items, and yields a score between 0 and 60, where higher scores indicates higher levels of depressive symptoms. A score of 16 or greater is used as the cut-off to indicate high levels of depressive symptoms.^36^

*Clinical data of patients*. Information about type of dementia, time since diagnosis of dementia, treatment for dementia, and Global Deterioration Scale (GDS) score were collected.

**Professionals involved.** Data collection was carried out by neurologists suitably trained and experienced in the tests applied.

**Statistical methods.** Descriptive statistics was performed using proportions for categorical variables. The skewness and kurtosis test (*sktest*) was used to assess the normality of the distributions. Therefore, numerical variables were summarized as median, minimum and maximum values. These results are showed stratified into four groups according to severity of burden: little or no burden (Group 1), mild to moderate burden (Group 2), moderate to severe burden (Group 3), and severe burden (Group 4).

Chi-Square and Mann-Whitney tests for pairs of groups were used to compare demographic, medical and other characteristics between groups (Group 1 versus all others, Group 2 versus Groups 3 and 4, Group 3 versus Group 4). Scatter plots were employed to graphically display the variability of caregiver burden according to time since diagnosis, GDS, and time caregiving, by type of dementia.

The predictive value of the measured variables was studied by multiple regression analysis. For this purpose, the hierarchical forward stepwise technique was applied to identify the best model to explain the variability of ZBI value. All analyses were performed with a confidence level of 0.95 using STATA 12.0 (*Stata Corporation, College Station, Texas*) software.

**Ethical aspects.** This study was authorized by the research office of Clínica Internacional. The present study was approved by the Research Ethics Committee of the Universidad Privada San Martin de Porres. All participants gave their informed consent to participate in the study.

## RESULTS

**Descriptive statistics.** Participants in the study included 92 informal caregivers, comprising 39 from Clínica Internacional - Lima, 33 from Instituto Peruano de Neurociencias and 20 from Clínica Internacional - San Borja. The caregivers had elevated ZBI scores (median = 37.5; minimum value = 3; and maximum value = 74). Care-recipients were predominantly 69 years old or over (75%), with at least one year since diagnosis (75%), a medical diagnosis of Alzheimer's disease (73.9%), received treatment (84.8%), and obtained a GDS score of 5 or more (75%).

The majority of caregivers were 55.5 years old or over (75%), female (81.5%), married (83.7%), spouse of care-recipients (60.87%), with at least 10 years of education (75.0%) and one year as caregiver (75%). Additionally, caregivers reported reduced entertainment time (90.2%) and self-perception of impaired health (83.7%). Finally, many caregivers reported working (37.0%), reduced working time (44.6%), external support for caring (39.1%), and impact on the household economy (47.8%). However, 62.0% of caregivers had the support of a second caregiver. Detailed information by severity of burden is shown in Tables 1 and 2.

**Table 1:** Characteristics and test scores by severity of caregiver burden in 92 patients with dementia attended at the neurology consultancy of three memory centers.

		Degree of severity			
		Little or no burden (n=26) n (%)	Mild to moderate burden (n=23) n (%)	Moderate to severe burden (n=24) n (%)	Severe burden (n=19) n (%)
Age of patient, years^§^		75 (57-88)	76 (60-92)	76 (40-89)	73 (56-84)
Time since diagnosis, years^§^		1 (0-3)	2 (1-3)*	2 (1-3)*	2 (0-3)*
Dementia sub-type	Alzheimer’s dementiaz	18 (69.2%)	16 (69.6%)	21 (87.5%)	13 (68.4%)
	Vascular dementia	4 (15.4%)	4 (17.4%)	2 (8.3%)	3 (15.8%)
	Frontotemporal dementia	1 (3.9%)	1 (4.4%)	1 (4.2%)	3 (15.8%)
	Mixed dementia	3 (11.5%)	2 (8.7%)	0 (0.0%)	0 (0.0%)
Treatment, type	None	2 (7.7%)	4 (17.4%)	5 (20.8%)	3 (15.8%)
	Cholinesterase inhibitors	19 (73.1%)	8 (34.8%)	6 (25.0%)	6 (31.6%)
	Memantine	2 (7.7%)	4 (17.8%)	1 (4.8%)	2 (10.5%)
	Cholinesterase inhibitors plus memantine	3 (11.5%)	7 (30.4%)	12 (50.0%)	8 (42.1%)
Global Dementia Scale, points^§^		4 (3-6)	5 (3-5)*	5 (3-6)*	5 (3-6)*

§ Data are expressed as median (minimum and maximum values);

* p-value for the comparison with the little or no burden group;

** p-value for the comparison with the mild to moderate burden group; *** p-value for the comparison with the moderate to severe burden group.

**Table 2:** Characteristics and test scores by severity of burden in 92 caregivers of patients with dementia attended at the neurology consultancy of three memory centers.

		Degree of severity			
		Little or no burden (n=26)	Mild to moderate burden (n=23)	Moderate to severe burden (n=24)	Severe burden (n=19)
Sex of caregiver: female		23 (88.5%)	21 (91.3%)	18 (75.0%)	13 (68.4%)
Age of caregiver, years^§^		68 (43-80)	65 (38-76)	56 (36-76)	57 (50-76)
Civil status of caregiver: married		25 (96.2%)	16 (69.6%)*	18 (75.0%)*	18 (94.7%)
Education of caregiver, years^§^		11 (5-16)	14 (6-16)	10 (5-18)	10 (5-16)
Relationship with patient	Spouse	19 (73.1%)	14 (60.9%)	9 (37.5%)	14 (73.7%)
	Son or daughter	7 (26.9%)	7 (30.4%)	11 (45.8%)	2 (10.5%)
	Other	0 (0.0%)	2 (8.7%)	4 (16.7%)	3 (15.8%)
Time caregiving, years^§^		1 (0-2)	1 (0-3)	1 (1-3)*	2 (0-3)*
Currently working		4 (15.4%)	6 (26.1%)*	12 (50.0%)* **	12 (63.2%)* ** ***
Reduced working time		3 (11.5%)	12 (52.1%)*	14 (58.3%)* **	12 (63.2%)* ** ***
Impact on the household economy		4 (15.4%)	12 (52.2%)*	15 (62.5%)* **	13 (68.4%)* ** ***
Family support		7 (26.9%)	6 (26.1%)	11 (45.8%)*	4 (21.1%)
External support for caring		14 (53.89%)	11 (47.8%) *	7 (29.2%)* **	4 (21.1%)* ** ***
Reduced entertainment time		18 (69.3%)	23 (100.0%)*	23 (95.8%)*	19 (100.0%)*
Self-perception of impaired health		13 (50.0%)	21 (91.3%)*	24 (100.0%)*	19 (100.0%)*
Availability of second caregiver		5 (19.2%)	14 (60.9%)*	21 (87.5%)* **	17 (89.5%)* ** ***
Beck Depression Index, points^§^		8 (4-19)	19 (7-25)*	29 (9-39)* **	34 (21-41)* **
Consequences of care on caregiver (S1), points		6 (0-14)	16 (10-30)*	27 (18-36)* **	33 (28-37)* ** ***
Beliefs and expectations about ability to care (S2), points		4 (0-9)	8 (0-14)*	15.5 (8-21)* **	21 (16-24)* ** ***
Relationship between caregiver and patient (S3), points		2 (0-5)	6 (4-11)*	10 (5-15)* **	13 (10-16)* ** ***

§ Data are expressed as median (minimum and maximum values);

* p-value for the comparison with the little or no burden group;

** p-value for the comparison with the mild to moderate burden group;

*** pvalue for the comparison with the moderate to severe burden group; S1-S3: Sub-tests of the Zarit test.

**Figure 1:**
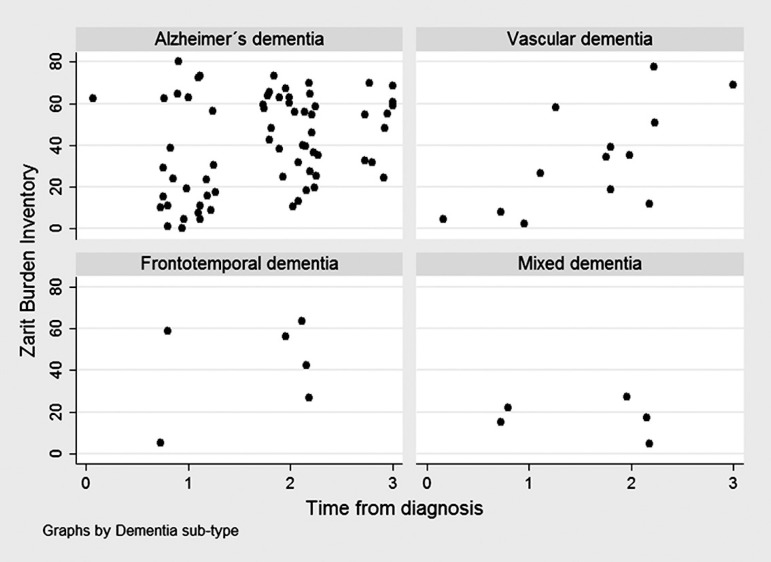
Zarit test scores according to time since diagnosis in 92 subjects attended at the neurology consultancy of the "Clínica Internacional", by dementia sub-type.

**Figure 2:**
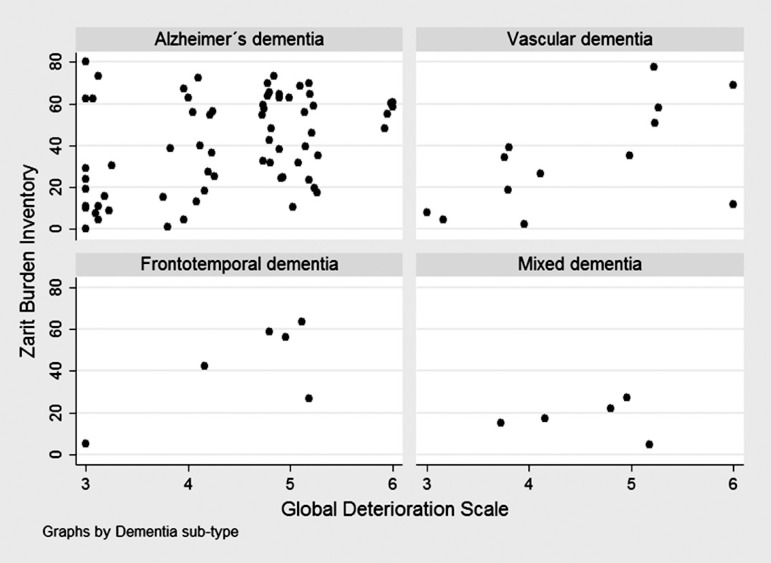
Zarit test scores according to global dementia scale points in 92 subjects attended at the neurology consultancy of "Clínica Internacional", by dementia sub-type.

**Figure 3:**
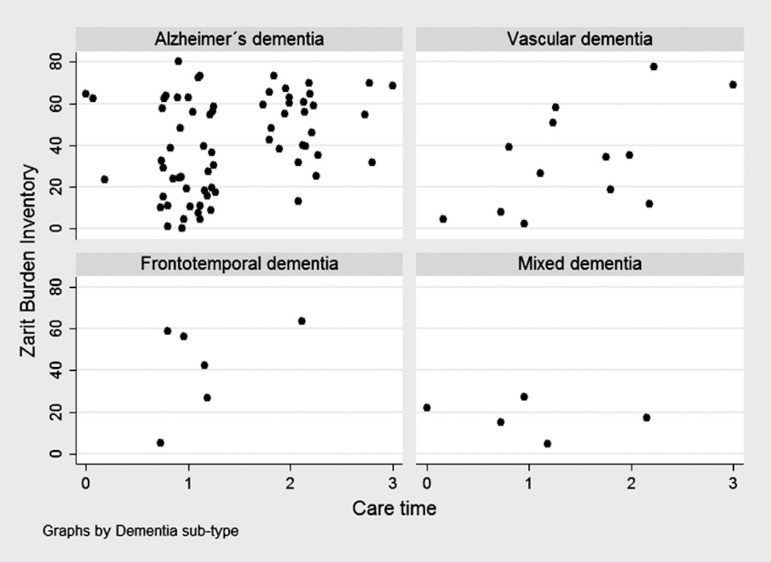
Zarit scores according to time caregiving in 92 subjects attended at the neurology consultancy of the "Clínica Internacional", by dementia sub-type.

The scatter plot showed no clear tendency of caregiver burden according to time since diagnosis (Figure 1), GDS (Figure 2), or time caregiving (Figure 3), particularly for Alzheimer's and mixed dementia sub-types.

**Analytical statistics.** Neither demographic nor clinical characteristics of patients with dementia were clearly associated with caregiver burden. Only time since diagnosis and GDS were significantly higher in caregivers with moderate severity burden or greater than in caregivers with little or no burden (Table 1).

None of the demographic characteristics of caregivers or existence of family support was associated with burden severity. Median time caregiving was significantly higher in caregivers with moderate burden severity or greater. The proportion of caregivers reporting reduced entertainment time and perception of impaired health was found to increase directly with burden severity (Table 2).

Working status (currently working and reduced working time), availability of a second caregiver and impact on household economy exhibited a significant, linear, positive association with burden severity in caregivers. External support in caring showed a significant, linear negative association with burden severity. Sub-tests of the ZBI were linearly positively associated with burden severity in caregivers (Table 2).

Finally, a multivariate model was built to predict ZBI values. According to statistical procedures, this hierarchical model (Adjusted R-squared = 0.7421) included the following variables (sorted by relevance for higher burden severity):

[1] self-perception of impaired health (β=7.16);
2) time caregiving (β_3_=5.69; β_2_= -1.43; and β_1_=-3.04);
[3] impact on family economy (β=4.71); GDS (β=1.58); currently working (β=1.42); BDI (β=1.38); reduced entertainment time (β=0.75); reduced working time (β= -0.18); family support (β= -2.39); and existence of other support (β= -5.41).

However, only BDI was statistically significant (p-value <0.001) (Table 3).

**Table 3:** Multivariate model to predict ZBI values in 92 caregivers of patients with dementia attended at the neurology consultancy of three memory centers.

		Adjusted R-squared = 0.7421	
		β	p-value
Self-perception of impaired health		7.156	0.086
Time caregiving^§^	Between 1 and 3 years	–3.046	0.597
	Between 3 and 6 years	–1.425	0.813
	Between 6 and 9 years	5.687	0.452
Impact on family economy		4.705	0.140
Currently working		1.582	0.667
Global Dementia Scale, points		1.425	0.375
Beck Depression Inventory, points		1.376	0.000
Reduced entertainment		0.747	0.874
Reduced working time		–0.181	0.965
Family support		–2.39	0.449
External support for caring		–5.411	0.078

§ Less than 1 year as reference; β: coefficient of regression; p-value for Wald test in multiple regression model.

## DISCUSSION

This study showed considerable levels of burden in a sample of Peruvian informal caregivers, higher than levels previously reported in other studies including Peruvian sub-samples.^37^ Additionally, we found that BDI-II value was a consistent predictor of ZBI value. However, self-perception of worsened health, repercussion on the family economy and time caregiving proved stronger determinants of ZBI than BDI-II value and caregiver working status or patient deterioration status.

Subjective burden has been defined as the attitudes and emotional reactions to the experience of caring and relates to how the situation is perceived, including difficulties and rewards. By contrast, objective burden refers to the performance of the caregiver and determinants of demands caring for patients, such as activities for caring, time spent, physical burden, and exposure to stressful situations related to caring needs.^38,39^ In our study, we assessed the subjective and objective dimension of the burden by means of the ZBI and questionnaire plus BDI-II, respectively.

The informal caregivers showed impairments in physical, psychological, and social performance,^38,40^ with perception of poor health and development of several medical problems and comorbidities.^41^ Furthermore, as well as the social and financial implications, caregivers suffer higher levels of depression and anxiety, greater incidence of physical health problems, and shortened life expectancy.^46^ A previous report highlighted that informal care was associated with psychiatric conditions (40% to 75%) such as depression (15% to 32%).^47^ In our study, at least 75% of caregivers had depression according to BDI values.

A recent systematic review suggested that caregiver characteristics have a greater impact on perceived burden, stress, and depression than the intensiveness of the care needed.^48^ However, relevant characteristics such as time caregiving or family support were not associated with ZBI values in our sample. This result could be related to limitations in measuring these variables since, among other factors, the commencement of caregiving is hard to define because it evolves naturally from support given and received normally before the onset of dementia and may precede or follow a formal diagnosis.^49^

We observed that the existence of several sources of support (family support, external support, and availability of a second caregiver) were not sufficient to counteract the burden of the caregiver. Similarly, previous studies have shown that caregiving usually does not cease even after admission to a nursing home because caregivers visiting their relatives are still involved in caring. Thus, the burden may persist after nursing home admission.^50^

A complete assessment of caregiver status should include the caregiver (burden and impacts) and care-recipients (health and non-health related problems).^51^ The relationship between informal caregivers and care-recipients influences the perceived impact of caring^48^ and the family is the main source of caring for patients with dementia.^52^ In our study, the majority of caregivers were spouses and, secondarily, son or daughter.

We highlighted that self-perception of impaired health, impact on the family economy and time caregiving care were the strongest determinants of caregiver burden. These findings are preliminary and prospective studies assessing the determinants of burden in caregivers should be conducted.

Our study has some limitations. We did not previously investigate the validity of the ZBI. The ZBI does not assess all measures of caregiver status, such as quality of life and we did not apply tests for these purposes.

We did not measure some variables such as living together (living with care-recipients), overburden (existence of other care-recipients, including children), details of caring (average time spent caring each day, techniques for caring, care training, living arrangements, and co-existence of professional service or support) and disease (perception of cognitive and behavioral impairments by patient and caregiver, complete assessment of the clinical status of patient and treatment compliance).

In summary, our sample of Peruvian informal caregivers showed elevated ZBI scores. The self-perception of worsened health, repercussion on family economy and time caregiving were the main determinants of ZBI, although only BDI was a consistent predictor of ZBI.

ZBI is an essential tool for measuring the burden of informal caregivers that combines several aspects of the impact on caregivers associated with the caring process. Thus, its use in clinical practice is valuable.

## References

[1] Cuijpers P (2005). Depressive disorders in caregivers of dementia patients: a
systematic review. Aging Ment Health.

[2] Pinquart M, Sörensen S (2003). Associations of stressors and uplifts of care-giving with
caregiver burden and depressive mood: a meta-analysis. J Gerontol B Psychol Sci Soc Sci.

[3] Brodaty H, Donkin M (2009). Family caregivers of people with dementia. Dialogues Clin Neurosci.

[4] Etters L, Goodall D, Harrison BE (2008). Caregiver burden among dementia patient caregivers: a review of
the literature. J Am Acad Nurse Pract.

[5] Lingler JH, Martire LM, Schulz R (2005). Caregiver-specific outcomes in anti-dementia clinical drug
trials: a systematic review and meta-analysis. J Am Geriatr Soc.

[6] Moniz-Cook E, Vernooij-Dassen M, Woods R, Verhey F, Chattat R, De Vugt M (2008). A European consensus on outcome measures for psychosocial
intervention research in dementia care. Aging Ment Health.

[7] Harvey K, Catty J, Langman A, Winfield H, Clement S, Burns E (2008). A review of instruments developed to measure outcomes for carers
of people with mental health problems. Acta Psychiatr Scand.

[8] Brodaty H (2007). Meaning and measurement of caregiver outcomes. Int Psychogeriatr IPA.

[9] Deeken JF, Taylor KL, Mangan P, Yabroff KR, Ingham JM (2003). Care for the caregivers: a review of self-report instruments
developed to measure the burden, needs, and quality of life of informal
caregivers. J Pain Symptom Manage.

[10] Dunkin JJ, Anderson-Hanley C (1998). Dementia caregiver burden: a review of the literature and
guidelines for assessment and intervention. Neurology.

[11] George LK, Gwyther LP (1986). Caregiver well-being: a multidimensional examination of family
caregivers of demented adults. Gerontologist.

[12] Montgomery RV, Stull DE, Borgatta EF (1985). Measurement and the analysis of burden. Res Aging.

[13] Losada A, Márquez-González M, Romero-Moreno R, López J (2014). Development and validation of the Experiential Avoidance in
Caregiving Questionnaire (EACQ). Aging Ment Health.

[14] Roach L, Laidlaw K, Gillanders D, Quinn K (2013). Validation of the Caregiver Guilt Questionnaire (CGQ) in a sample
of British dementia caregivers. Int Psychogeriatr IPA.

[15] Stephan A, Mayer H, Renom Guiteras A, Meyer G (2013). Validity, reliability, and feasibility of the German version of
the Caregiver Reaction Assessment scale (G-CRA): a validation
study. Int Psychogeriatr IPA.

[16] Erder MH, Wilcox TK, Chen W-H, O'Quinn S, Setyawan J, Saxton J (2012). A new measure of caregiver burden in Alzheimer's disease: the
caregiver-perceived burden questionnaire. Am J Alzheimers Dis Other Demen.

[17] Rosness TA, Haugen PK, Gausdal M, Gjøra L, Engedal K (2012). Carers of patients with early-onset dementia, their burden and
needs: a pilot study using a new questionnaire-care-EOD. Int J Geriatr Psychiatry.

[18] Guerra-Silla MG, Gutiérrez-Robledo LM, Villalpando-Berumen JM (2011). Psychometric evaluation of a Spanish language version of the
Screen for Caregiver Burden (SCB) in caregivers of patients with mixed,
vascular and Alzheimer's dementia. J Clin Nurs.

[19] Salvia MG, Dawidowski A, Schapira M (2011). Spanish Revised Memory and Behavior Problems Checklist Scale
(SpRMBPC): trans-cultural adaptation and validation of the RMBPC
questionnaire. Int Psychogeriatr IPA.

[20] Seng BK, Luo N, Ng WY (2010). Validity and reliability of the Zarit Burden Interview in
assessing caregiving burden. Ann Acad Med Singapore.

[21] Gómez-Ramos MJ, González-Valverde FM (2004). El cuidador del paciente con demência: aplicación
del test Índice del Esfuerzo del Cuidador. Rev Esp Geriatr Gerontol.

[22] Zarit SH, Reever KE, Bach-Peterson J (1980). Relatives of the impaired elderly: correlates of feelings of
burden. Gerontologist.

[23] Martín-Carrasco M, Domínguez-Panchón AI, Muñoz-Hermoso P, González-Fraile E, Ballesteros-Rodríguez J (2013). Instrumentos para medir la sobrecarga en el cuidador informal del
paciente con demencia. Rev Esp Geriatr Gerontol.

[24] Merino-Soto C, Angulo-Ramos M (2013). Validación en Chile de la escala de sobrecarga del
cuidador de Zarit en sus versiones original y abreviada:
corrección. Rev Médica Chile.

[25] Arai Y (2011). [The Japanese version of the Zarit Caregiver Burden
Interview]. Nihon Rinsho Jpn J Clin Med.

[26] Braun M, Scholz U, Hornung R, Martin M (2010). The burden of spousal caregiving: a preliminary psychometric
evaluation of the German version of the Zarit burden
interview. Aging Ment Health.

[27] Breinbauer K H, Vásquez V H, Mayanz S S, Guerra C, Millán K T (2009). Validación en Chile de la Escala de Sobrecarga del
Cuidador de Zarit en sus versiones original y abreviada. Rev Médica Chile.

[28] Taub A, Andreoli SB, Bertolucci PH (2004). Dementia caregiver burden: reliability of the Brazilian version
of the Zarit caregiver burden interview. Cad Saude Publica.

[29] Marín M (1996). Adaptación para nuestro medio de la Escala de Sobrecarga
del Cuidador de Zarit. Rev Multidiscip Gerontol.

[30] Brown LJ, Potter JF, Foster BG (1990). Caregiver burden should be evaluated during geriatric
assessment. J Am Geriatr Soc.

[31] Rankin ED, Haut MW, Keefover RW, Franzen MD (1994). The establishment of clinical cutoffs in measuring caregiver
burden in dementia. Gerontologist.

[32] Wang Y-P, Gorenstein C (2013). Psychometric properties of the Beck Depression Inventory-II: a
comprehensive review. Rev Bras Psiquiatr.

[33] Segal DL, Coolidge FL, Cahill BS, O'Riley AA (2008). Psychometric properties of the Beck Depression Inventory II
(BDI-II) among community-dwelling older adults. Behav Modif.

[34] Steer RA, Rissmiller DJ, Beck AT (2000). Use of the Beck Depression Inventory-II with depressed geriatric
inpatients. Behav Res Ther.

[35] Wiebe JS, Penley JA (2005). A psychometric comparison of the Beck Depression Inventory-II in
English and Spanish. Psychol Assess.

[36] Beck AT, Steer RA, Ball R, Ranieri W (1996). Comparison of Beck Depression Inventories -IA and -II in
psychiatric outpatients. J Pers Assess.

[37] Prince M, Brodaty H, Uwakwe R, Acosta D, Ferri CP, Guerra M (2012). Strain and its correlates among carers of people with dementia in
low-income and middle-income countries. A 10/66 Dementia Research Group
population-based survey. Int J Geriatr Psychiatry.

[38] Montorio Cerrato I, Fernández de Trocóniz MI, López López A, Sánchez Colodrón M (1998). La Entrevista de Carga del Cuidador. Utilidad y validez del
concepto de carga. Anales de Psicología.

[39] Del Mar García-Calvente M, Mateo-Rodríguez I, Maroto-Navarro G (2004). Gac Sanit. SESPAS.

[40] Wu T, Lo K (2007). Healthy aging for caregivers: what are their
needs?. Ann N Y Acad Sci.

[41] Bridges-Webb C, Giles B, Speechly C, Zurynski Y, Hiramanek N (2007). Patients with dementia and their carers. Ann N Y Acad Sci.

[42] Hoskins S, Coleman M, McNeely D (2005). Stress in carers of individuals with dementia and Community
Mental Health Teams: an uncontrolled evaluation study. J Adv Nurs.

[43] Schulz R, Patterson TL (2004). Caregiving in geriatric psychiatry. Am J Geriatr Psychiatry.

[44] Hébert R, Lévesque L, Vézina J (2003). Efficacy of a psychoeducative group program for caregivers of
demented persons living at home: a randomized controlled
trial. J Gerontol B Psychol Sci Soc Sci.

[45] Patterson TL, Grant I (2003). Interventions for caregiving in dementia: physical
outcomes. Curr Opin Psychiatry.

[46] Schulz R, Martire LM, Klinger JN (2005). Evidence-based caregiver interventions in geriatric
psychiatry. Psychiatr Clin North Am.

[47] Consortium ADI (2009). World Alzheimer Report.

[48] Schoenmakers B, Buntinx F, Delepeleire J (2010). Factors determining the impact of care-giving on caregivers of
elderly patients with dementia. A systematic literature. Maturitas.

[49] Gaugler JE, Zarit SH, Pearlin LI (2003). The onset of dementia caregiving and its longitudinal
implications. Psychol Aging.

[50] Gaugler JE, Mittelman MS, Hepburn K, Newcomer R (2010). Clinically significant changes in burden and depression among
dementia caregivers following nursing home admission. BMC Med.

[51] Poulshock SW, Deimling GT (1984). Families caring for elders in residence: issues in the
measurement of burden. J Gerontol.

[52] Eurostat 2003 (2013). Feasibility Study - Comparable Statistics in the Area of Care of
Dependent Adults in the European Union.

